# Automatic Vertebral Body Segmentation Based on Deep Learning of Dixon Images for Bone Marrow Fat Fraction Quantification

**DOI:** 10.3389/fendo.2020.00612

**Published:** 2020-09-02

**Authors:** Jiamin Zhou, Pablo F. Damasceno, Ravi Chachad, Justin R. Cheung, Alexander Ballatori, Jeffrey C. Lotz, Ann A. Lazar, Thomas M. Link, Aaron J. Fields, Roland Krug

**Affiliations:** ^1^Department of Radiology and Biomedical Imaging, School of Medicine, University of California, San Francisco, San Francisco, CA, United States; ^2^Bakar Computational Health Sciences Institute, University of California, San Francisco, San Francisco, CA, United States; ^3^Center for Intelligent Imaging, University of California, San Francisco, San Francisco, CA, United States; ^4^Department of Orthopaedic Surgery, School of Medicine, University of California, San Francisco, San Francisco, CA, United States; ^5^Department of Preventive and Restorative Dental Sciences, School of Dentistry, University of California, San Francisco, San Francisco, CA, United States; ^6^Department of Epidemiology and Biostatistics, School of Medicine, University of California, San Francisco, San Francisco, CA, United States

**Keywords:** spine imaging, bone marrow fat, biomarkers, deep learning, segmentation, magnetic resonance imaging

## Abstract

**Background:** Bone marrow fat (BMF) fraction quantification in vertebral bodies is used as a novel imaging biomarker to assess and characterize chronic lower back pain. However, manual segmentation of vertebral bodies is time consuming and laborious.

**Purpose:** (1) Develop a deep learning pipeline for segmentation of vertebral bodies using quantitative water-fat MRI. (2) Compare BMF measurements between manual and automatic segmentation methods to assess performance.

**Materials and Methods:** In this retrospective study, MR images using a 3D spoiled gradient-recalled echo (SPGR) sequence with Iterative Decomposition of water and fat with Echo Asymmetry and Least-squares estimation (IDEAL) reconstruction algorithm were obtained in 57 subjects (28 women, 29 men, mean age, 47.2 ± 12.6 years). An artificial network was trained for 100 epochs on a total of 165 lumbar vertebrae manually segmented from 31 subjects. Performance was assessed by analyzing the receiver operating characteristic curve, precision-recall, F1 scores, specificity, sensitivity, and similarity metrics. Bland-Altman analysis was used to assess performance of BMF fraction quantification using the predicted segmentations.

**Results:** The deep learning segmentation method achieved an AUC of 0.92 (CI 95%: 0.9186, 0.9195) on a testing dataset (*n* = 24 subjects) on classification of pixels as vertebrae. A sensitivity of 0.99 and specificity of 0.80 were achieved for a testing dataset, and a mean Dice similarity coefficient of 0.849 ± 0.091. Comparing manual and automatic segmentations on fat fraction maps of lumbar vertebrae (*n* = 124 vertebral bodies) using Bland-Altman analysis resulted in a bias of only −0.605% (CI 95% = −0.847 to −0.363%) and agreement limits of −3.275% and +2.065%. Automatic segmentation was also feasible in 16 ± 1 s.

**Conclusion:** Our results have demonstrated the feasibility of automated segmentation of vertebral bodies using deep learning models on water-fat MR (Dixon) images to define vertebral regions of interest with high specificity. These regions of interest can then be used to quantify BMF with comparable results as manual segmentation, providing a framework for completely automated investigation of vertebral changes in CLBP.

## Introduction

Bone marrow fat (BMF) content in the vertebral bodies was found to correlate with disease severity in patients with osteoporosis and HIV, and was associated with chronic low back pain (CLBP); it therefore may provide a potential imaging biomarker for patients with various skeletal, metabolic, and hematological diseases both to assess disease burden and monitor treatment (1–3). For example, increases in vertebral BMF and BMF heterogeneity could play a role in the etiology of intervertebral disc degeneration (3), which is often linked to CLBP, and alterations in vertebral BMF may accompany painful, innervated bone marrow lesions (BML) in the endplate (4–6). Yet, despite the potential importance of vertebral BMF as an imaging biomarker, clinical spine imaging relies mainly on qualitative interpretations based on T1- and T2-weighted images.

Recent advances in quantitative MRI techniques enable accurate BMF measurement. For example, accurate assessment of BMF in the presence of trabecular bone can be achieved through chemical shift encoding-based water-fat imaging (7, 8), or the Dixon method, allowing for the ability to establish relationships between changes in BMF occurring in diseases such as CLBP. In water-fat imaging, multiple echoes in a gradient echo acquisition are used to create a time-dependent phase shift between water and fat MR signals (9). Strong correlations have been shown between bone marrow fat fractions in lumbar vertebral bodies obtained from modified Dixon sequences and those obtained from single-voxel magnetic resonance spectroscopy (MRS), which is considered a gold standard method for quantification of bone marrow fat (10, 11). The iterative decomposition of water and fat with echo asymmetry and least-squares estimation (IDEAL) technique has also been developed through further modifications of the Dixon technique to overcome field inhomogeneities during water-fat separation with maximal signal-to-noise ratio (SNR) and minimal scanning time (12).

Although quantitative methods for BMF measurement are promising, BMF analysis from the resulting images requires manual identification of vertebral bodies and segmentation of regions of interest which, in MR images, is laborious and time consuming. One potential solution is deep learning, which has demonstrated robust automated segmentation performance in various biomedical imaging problems (13–16). Given large amounts of data, deep learning algorithms, mostly in the form of convolutional neural networks (CNNs), can automatically learn and thus predict representative features for a given medical imaging problem. Advantages of a machine learning model include the ability to review large amounts of data and consistently and objectively arrive at the same result without fatigue, as well as find nuance in images that may be difficult to detect by humans. Previous studies have looked into the feasibility of using deep learning, namely CNNs, to segment vertebrae in a two-step process including initial detection of the vertebrae (17–20). The U-Net deep learning architecture, which involves a contracting downsampling path and an expansive upsampling and concatenation path, has been proven to be effective in biomedical image segmentation tasks even with limited data availability (13). Some recent studies have used the U-Net for segmenting vertebrae in spinal CT and on sagittal and axial T2-weighted spine MRI (21–23), but so far U-Nets haven't been used for segmenting vertebrae in water-fat Dixon MR images, which typically have lower resolution and higher sensitivity to field inhomogeneities.

Given the potential value of quantitative BMF analysis in evaluating and monitoring disease activity, this study aimed to develop and evaluate a fully automated deep learning pipeline from water-fat Dixon MR images using U-Net to segment lumbar vertebral bodies on BMF maps in patients with CLBP and healthy controls. We hypothesized that the deep learning-based automated vertebral body segmentation will allow for a faster workflow and comparable accuracy in both region of interest (ROI) segmentation and BMF quantification thereafter.

## Materials and Methods

### Data Selection and Study Design

Full Institutional Review Board approval and written informed consent was obtained from each subject in this prospective study. The dataset used in this study consisted of 57 subjects enrolled between January 2016 and July 2018, with a mean age of 47.2 ± 12.6 years. Male and non-pregnant female patients between 18 and 70 years of age were included. Twenty-eight of the participants were female (49.1%). Forty subjects had been experiencing low back pain more than three consecutive months, with a back-pain score ≥30% on the Oswestry disability index (ODI) or ≥4 on the visual analog scale (VAS). Seventeen subjects were healthy controls without low back pain (VAS ≤1). Subjects were excluded if they had diabetes, smoking, cancer, and lumbar vertebral abnormalities (spondylolisthesis, spondylolysis, lumbar scoliosis, lumbar disc herniation). A subset of these subjects was included in previous studies (3, 24). Detailed demographics are described in Table 1.

**Table 1:** Demographic characteristics of the dataset the deep learning model was trained on, and the two test sets used for evaluation.

**Characteristic**	**Set 1 (*n* = 31)**	**Set 2 (*n =* 11)**	**Set 2&3 (*n* = 26)**
Age (years)	47.9 ± 12.4	50.7 ± 9.7	46.4 ± 12.9
Sex			
Female	16 (51.6)	2 (18.2)	12 (46.2)
Male	15 (48.4)	9 (81.8)	14 (53.8)
Patient Status			
Controls	8 (25.8)	1 (9.1)	9 (34.6)
Cases	23 (74.2)	10 (90.9)	17 (65.4)
Weight (kg)	73.7 ± 15.8	87.4 ± 16.5	77.2 ± 16.8
Height (cm)	173.4 ± 8.5	177.0 ± 10.5	174 ± 11.7
BMI (kg/m^2^)	24.4 ± 4.4	28.0 ± 5.5	25.7 ± 5.1
Clinical measures			
ODI	24.6 ± 19.7	32.7 ± 17.6	20.8 ± 19.0
VAS	4.9 ± 3.4	6.1 ± 2.3	4.2 ± 3.2

–*Mean data are ± standard deviation; data in parentheses are percentages. Set 1 was the IDEAL image dataset used to train the U-Net. Set 2&3 consists of the 11 subjects in Set 2 and 15 additional subjects scanned after July 2017. BMI, body mass index; ODI, Oswestry Disability Index; VAS, Visual Analog Scale*.

### MRI Acquisition

Magnetic resonance images were obtained using a Discovery MR 750 3T scanner with an 8-channel phased-array spine coil (GE Healthcare, Waukesha, WI). Clinical fast spin echo (FSE) sagittal images with T1 and T2 weighting were acquired with field of view (FoV) = 26 cm and slice thickness = 4 mm. T2-weighted FSE had an echo time (TE) = 60 ms, repetition time (TR) = 4877 ms, echo train length (ETL) = 24, readout-bandwidth (rBW) = ±50 kHz, and in-plane resolution of 0.6 mm. T1-weighted FSE had TE = 30 ms, TR = 511 ms, ETL = 4, rBW = ±50 kHz, and in-plane resolution of 0.5 mm. The water-fat MRI protocol consisted of a sagittal 3D spoiled gradient-recalled echo (SPGR) sequence with six echoes (TE_1_ = 2.1 ms, TE_2_ = 3.1 ms, TE_3_ = 4.1 ms, TE_4_ = 5.1 ms, TE_5_ = 6.1 ms, and TE_6_ = 7.0 ms) and iterative decomposition of water and fat with echo asymmetry and least-squares estimation (IDEAL) reconstruction algorithm (25) with TR = 7 ms, TE = 2.1 ms, flip angle = 3°, rBW = ±83.3 kHz, FoV = 22 cm, in-plane resolution = 1.375 mm, and slice thickness = 4 mm. Each subject had 20 slices in each IDEAL series (water, fat, ${\text{R}}_{2}^{*}$, and fat fraction map). The generated water-only, fat-only, ${\text{R}}_{2}^{*}$ images as well as the fat fraction maps were transferred from the scanner to a Linux workstation for the subsequent analyses. All IDEAL image series contained 5-7 mid-sagittal slices with visible lumbar vertebrae, and sometimes parts of the T12 vertebral body and/or sacrum.

### Manual Vertebral Body Segmentation and Inter-Rater Reliability

Two sets of manual segmentations for all five lumbar vertebral bodies were performed independently by two different trained operators (J.R.C. and A.B.) with different guidelines, both using in-house software developed with an interactive display language routine (IDL, Harris Geospatial Solutions, Broomfield, CO). Rater A (J.R.C.), under supervision by a musculoskeletal imaging fellowship-trained radiologist (T.M.L.), performed segmentations on seven mid-sagittal slices of water IDEAL images for 42 subjects (CLBP = 33, Controls = 9) scanned prior to August 2017. Rater B (A.B.), under supervision of a spine imaging expert with 10 years of experience reading spine MRIs (A.J.F.), performed segmentations on five mid-sagittal slices of the derived fat fraction maps for 57 subjects (CLBP = 40, Controls = 17), including all 42 subjects segmented by Rater A, whilst using the T1 series acquired during the scanning session as a guide. Contours were drawn up to but not including the thick margins of cortical bone separating vertebral body from surrounding tissues. Segmentations were performed on 2x magnified images.

Cohen's kappa (κ) was used to test for interrater agreement between the two annotators on classifying whether a pixel from an IDEAL imaged belonged to the vertebra or non-vertebra class. The kappa is defined as

(1) $$\begin{array}{l} \kappa =\frac{p_{o}-p_{e}}{1-p_{e}} \end{array}$$

where *p*_*o*_ is the observed agreement ratio and *p*_*e*_ is the expected agreement ratio when both annotators independently assign labels by chance (26). This chance agreement is obtained through a per-annotator empirical prior over the class labels (27).

### Deep Learning

Preprocessing, deep learning model implementation, and model evaluation were performed in Python 3.7 (open-source; Python Software Foundation, Wilmington, DE) in a 12-core/24-thread AMD Ryzen Threadripper 1920X processor at 4.0 GHz (Advanced Micro Devices, Santa Clara, CA), 32 GB DDR4-SDRAM and a Titan Xp 11 GB graphical processing unit (Nvidia, Santa Clara, CA) running Linux system (Ubuntu 16.04; Canonical, London, England) with CUDA 10.0 (Nvidia).

A U-Net was trained using Keras with Tensorflow backend on a random selection of 31 subjects (CLBP = 23, Controls = 8), 7 of which were set aside for validation during the training process to prevent overfitting (13, 28). A Jaccard distance loss was used with Adam optimization, learning rate = 1e-4, and batch size = 4 with early stopping implemented (14, 29). Code can be found online (*https://github.com/zhoji/verteseg*). The ground truth segmentations for training the model was created manually by Rater A as described in section Manual Vertebral Body Segmentation and Inter-rater Reliability.

For each subject, twenty 256 × 256 slices (unchanged from original resolution) were input into an unchanged U-Net with default parameters, each image containing four “channels” corresponding to the water, fat, fat fraction, and R2^*^ values. No cropping was performed, and data were not normalized and kept at their original signal intensity values. The U-Net performance was evaluated on two separate testing sets. The first testing set (Set 2A) consisted of 11 subjects (CLBP = 10, Controls = 1) with labels annotated by rater A. The other testing set (Set 2B&3B) consisted of 26 subjects (CLBP = 17, Controls = 9), including the 11 subjects in the Set 2A, with labels annotated by rater B.

### ROI Quantification

**Figure 1:**
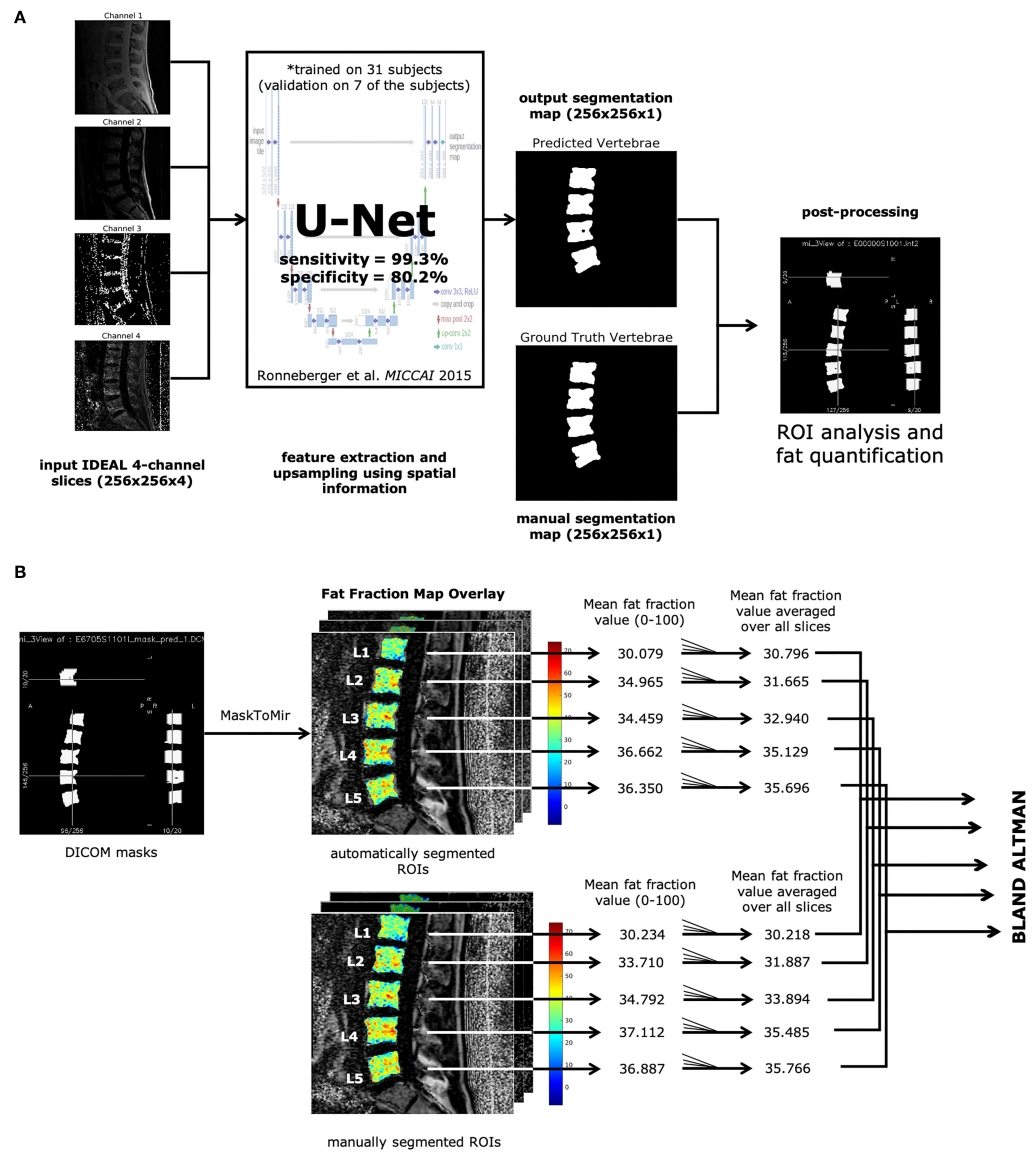
Automatic vertebrae segmentation and fat quantification pipeline. **(A)** All IDEAL images (water, fat, fat fraction, and ${\text{R}}_{2}^{*}$) are fed into a U-Net (13) as multichannel inputs, resulting in the predicted segmentation map. Each ROI corresponding to lumbar vertebrae was analyzed on fat fraction maps to yield mean BMF values. **(B)** DICOM masks were made from the predicted segmentation map. ROIs were identified through the MaskToMir function in in-house software made in IDL (IDL, Research Systems, Broomfield, CO). These automatically identified ROIs were then overlaid on the fat fraction maps derived from the water-fat IDEAL image series. For each lumbar vertebral body ROI, the mean fat fraction value was obtained for each slice, and the final mean BMF was averaged over all slices with lumbar vertebral bodies present. The mean BMFs for each lumbar vertebral body as defined by the automatically segmented ROIs were compared with the manually segmented ROIs through Bland-Altman analysis.

Our overall pipeline for segmentation and analysis is shown in Figure 1A. Resulting predicted segmentations from the trained U-Net were binarized with a threshold of 0.5 and used to create segmentation masks in DICOM format. Initial regions of interest (ROIs) derived from these segmentation masks were automatically defined using a function from in-house software developed with IDL 8.4 (IDL, Research Systems, Broomfield, CO). ROIs in each slice that cover more than half the area of their corresponding lumbar vertebral body (L1-5) were included for BMF analysis. Bone marrow fat fractions were quantified by taking the mean value of the fat fraction map in each ROI on a slice-by-slice basis, then averaging for all slices for each lumbar vertebral body (L1-L5), as described in Figure 1B. The final mean BMFs for each lumbar vertebral body as defined by the segmentations from the U-Net were compared with manual segmentations by rater A and rater B through Bland-Altman analysis.

**Figure 2:**
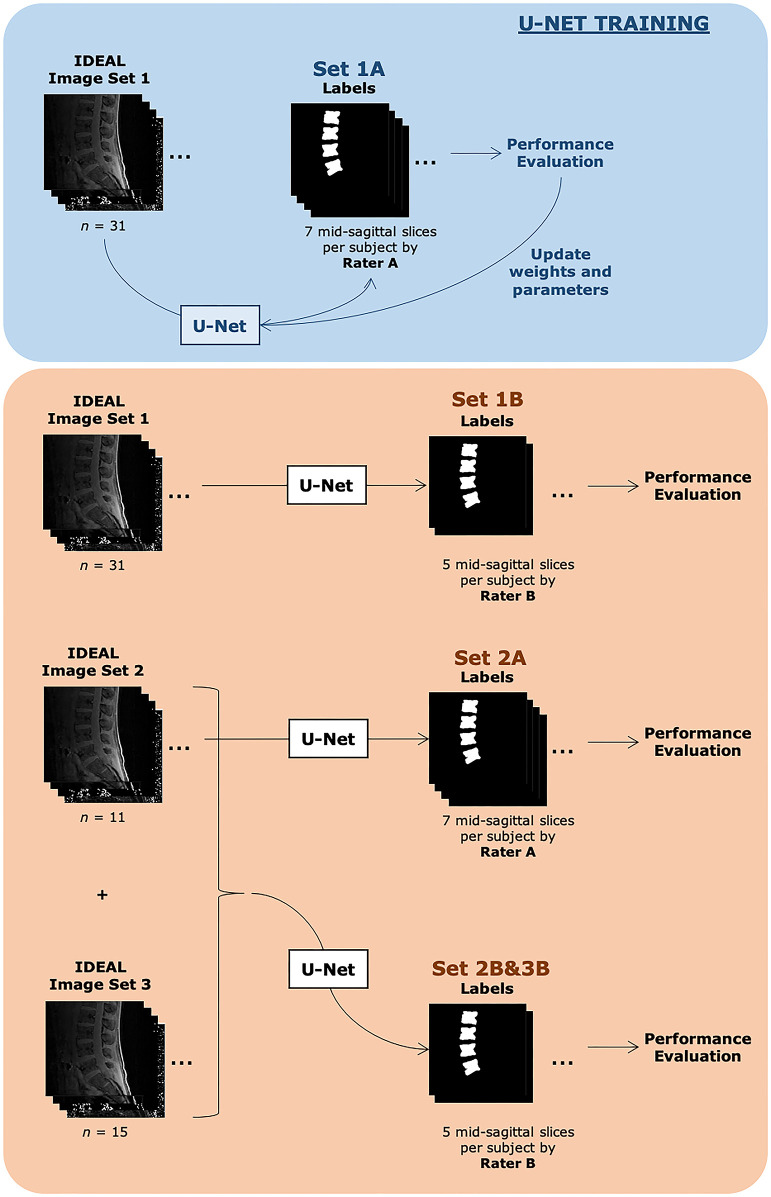
Description of datasets evaluated in this study. Both training sets consisted of the same 31 subjects. Set 1A was used to train the U-Net. Automatic segmentations from Set 1A were compared with manual segmentations done by Rater A and also used during the U-Net training process. The U-Net with finalized weights after training was then used to automatically segment images from the same image set 1 and compared with manual segmentations done by Rater B, denoted as Set 1B. Set 2A consisted of 11 subjects, with 7 slices each with identified vertebra from Rater A's manual segmentation, while Set 2B&3B consisted of 26 subjects, with 5 slices each with identified vertebra from Rater B's manual segmentation. Additional demographic information can be found in Table 1.

A diagrammatic representation of the training and testing datasets and associated labels is shown in Figure 2.

### Statistical Analysis

Final model performance was evaluated on both test sets on slices with vertebral segmentations present. Average script run-time was assessed over 10 runs using hyperfine (*github.com/sharkdp/hyperfine*). Model precision-recall, confusion matrices, and receiver-operating characteristic (ROC) curves, and Cohen κ values were calculated using scikit-learn 0.22.1 (*scikit-learn.org*). The DeLong algorithm was used to measure the uncertainty of an AUC, or area under the ROC curve (30). The F1-score and precision-recall curve AUC were also analyzed to better assess an imbalanced dataset (31). Dice similarity coefficients (DSC) and Jaccard similarity coefficients (also known as intersection over union, IoU) were also measured for slices that contained manually segmented vertebrae, and calculated as follows:

(2) $$\begin{array}{l} DSC=\frac{2|X\cap Y|}{\left|X|+|Y\right|}=\frac{2TP}{2TP+FP+FN} \end{array}$$

(3) $$\begin{array}{l} IoU=\frac{\left|X\cap Y\right|}{\left|X\cup Y\right|}=\frac{TP}{TP+FP+FN} \end{array}$$

where *X* and *Y* are the manual and predicted segmented masks, *TP* is the true positive count, *FP* is the false positive count, and *FN* is the false negative count.

Bland-Altman analysis was performed and visualized in Microsoft Excel 2019. Bland-Altman plots were created comparing the mean fat fractions in each vertebral body ROI as defined by the manual segmentations done for Test Set A and B, with those defined by the automatic segmentation (32). The limits of agreement (LOA) were calculated using the mean and the standard deviation (±1.96SD) of the differences between the two measurements for each lumbar vertebral body.

Intra-subject repeatability analysis was performed on a separate water-fat IDEAL MRI dataset of healthy controls (*n* = 8) acquired on a GE MR750w widebore MR 3T scanner with an 8-channel phased-array spine as a sagittal 3D SPGR sequence with six echoes and IDEAL reconstruction algorithm (20) with TR = 7 msec, FoV = 26 cm, and in-plane resolution = 1.3 mm. All other parameters were the same as those of the original dataset described in section MRI acquisition. For each scanning session, the subject exited the MRI, walked around for about 2 min, and then re-entered the MRI bore for a second water-fat IDEAL scan with the same parameters as before. Vertebral body segmentations were then inferred using the trained U-Net on the test and retest scans, and mean fat fraction for each lumbar vertebral body was quantified. Repeatability of the automated segmentation method on different scans of the same subject was assessed using a linear mixed model with random intercept to account for the repeated measured data. A repeated-measures ANOVA with a least mean-squares method was used to compare fat fraction percentage. A *post-hoc* Tukey-Kramer *t*-test was used to evaluate the difference in the first and second fat fraction percentages at each vertebral level taken from the scans in order to correct for multiple comparisons. An Intraclass Correlation Coefficient (ICC) assessed the reliability of the data (33). Short-term precision error was calculated to look at possible measurement error (34). A *p*-value < 0.05 was considered statistically significant. All repeatability analyses were completed in SAS (version 9.4) and Microsoft Excel 2019.

## Results

### Inter-Rater Reliability in Manually Segmented Images

Inter-rater reliability was assessed between the two annotators for their agreement on presence of absence of vertebra. The linearly weighted Cohen's kappa between the segmentations done by rater A and rater B was 0.798, consistent with a moderately strong level of agreement.

### Deep Learning Model Performance on Segmentation

After training the deep learning model for 100 epochs on 620 slices total (encompassing 155 lumbar vertebrae from 31 subjects), the U-Net accurately classified vertebral bodies in 97.8% of pixels in Sets 2A and 2B&3B. A sensitivity of 0.77 and specificity of 1.0 were achieved for Set 2A and a sensitivity of 0.80 and specificity of 0.99 were achieved for Set 2B&3B. An overview of the results with accuracy, precision, recall, F1 score, and sensitivity and specificity are shown in Supplementary Table 1.

With the use of the trained U-Net to classify vertebral bodies, the overall area under the receiver operating characteristic curve (AUC) was 0.99 (confidence interval [CI] 95%: 0.9861, 0.9864) on the training dataset Set 1A, 0.91 (CI 95%: 0.9058, 0.9071) on Set 2A, and 0.92 (CI 95%: 0.9186, 0.9195) on Set 2B&3B for classification of pixels as vertebrae. The receiver-operating characteristic curve is shown in Supplementary Figure 1A, while the precision-recall curve is shown in Supplementary Figure 1B. Precision-recall was analyzed due to better visual interpretability when dealing with imbalanced datasets (31).

We also analyzed the Dice similarity coefficient and intersection over union of the binarized predicted segmentations compared with both sets of manual segmentations for both the training set and the testing set. As expected, both DSC and IoU were very high for the predictions on the training set when compared with Rater A's ground truth manual segmentations, on which the neural network was trained (DSC: 0.959 ± 0.0756; IoU: 0.928 ± 0.105). On unseen data, the predicted segmentations for Sets 2A (DSC: 0.838 ± 0.198; IoU: 0.757 ± 0.216) and 2B&3B (DSC: 0.849 ± 0.091; IoU: 0.747 ± 0.118) still show moderately high agreement. Interestingly, when comparing the predicted segmentations for the training set with the manual segmentations done by Rater B (an independent manual segmentation that was not used as ground truth during model training), we see similar performance as the two test sets, as if the data were unseen (DSC: 0.886 ± 0.040; IoU: 0.798 ± 0.059), which suggests that the performance of the trained model is relatively insensitive to inter-rater differences in ground truth segmentations used to train the model. A summary of these agreement metrics is shown in Table 2.

**Table 2:** Metrics of agreement for manual and automatic segmentations in different datasets.

**Similarity metric**	**Set 1A**	**Set 1B**	**Set 2A**	**Set 2B&3B**
DSC	0.956 ± 0.076	0.886 ± 0.040	0.838 ± 0.198	0.849 ± 0.091
IoU	0.928 ± 0.105	0.798 ± 0.059	0.757 ± 0.216	0.747 ± 0.118

–*Mean data are ± standard deviation. Datasets used are as described in Figure 2. Model predictions for each slice were binarized by a threshold of >0.5. DSC, Dice Similarity Coefficient; IoU, Intersection over Union (Jaccard)*.

An added benefit of applying an automated method is increased time efficiency in the image analysis workflow compared with manual segmentation. With the deep learning model, running the script for segmentation of a series of spine images (twenty slices total, including five mid-sagittal slices with manual segmentations as reference) on a standard Linux workstation with 4 4-core/8-thread Intel Xeon W3550 processors at 3.07 GHz (Intel Corporation, Santa Clara, CA) and 12GB RAM, including loading input data and saving predictions, took a mean time of 16 ± 1 s (*n* = 10 runs; range: 14.5 to 19.1 s). It took about 5 min, on average, to manually segment all five lumbar vertebral bodies in a single slice, resulting in an acceleration of ~92x when using the automated method for five mid-sagittal slices.

### Bone Marrow Fat Fraction Quantification Performance

**Figure 3:**
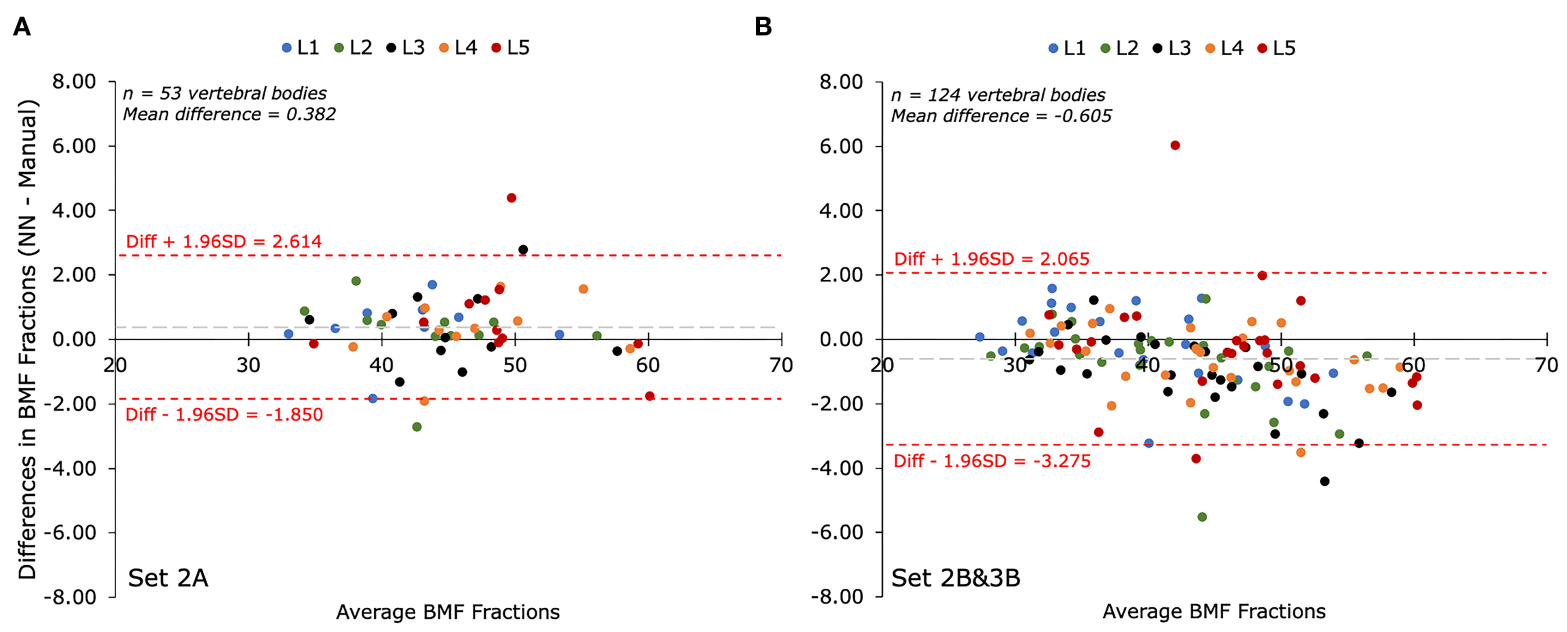
Bland-Altman plots of mean bone marrow fat fraction (BMF) percentages (%) as determined by manual segmentation compared to automatic segmentation for each lumbar vertebral body (L1-L5). The biases between BMFs collected by the automated (NN) and manual segmentations for both test sets were less than 10% of the mean value. **(A)** Comparison of mean BMF values for manual segmentations performed by annotator A and the predicted segmentation by the deep learning model on Set 2A (*n* = 53 vertebrae). The bias was +0.382% with limits of agreement of −1.850% and +2.614%. **(B)** Comparison of mean BMF values for manual segmentations performed by annotator B and the predicted segmentation by the deep learning model on Set 2B&3B (*n* = 124 vertebrae). The bias was−0.605% with limits of agreement of −3.275% and +2.065%.

The performance of the automatic segmentation on creating regions of interest for the purpose of calculating BMF was assessed with Bland-Altman analysis for the two testing datasets compared to their respective manual segmentations. Comparing manual and automatic segmentations on fat fraction maps using Bland-Altman analysis results in a positive bias (mean: +0.382%; CI 95% = +0.068 to 0.696%) in Set 2A (*n* = 53 vertebral bodies), as seen in Figure 3A. A total of 5.7% of the residuals fell outside the 1.96 ± SD (−1.850 to +2.614%) limits of agreement. For Set 2B&3B (*n* = 124 vertebral bodies), there was a negative bias (mean: −0.605%; CI 95% = −0.847 to −0.363%) with a total of 2.4% of the residuals falling outside the 1.96 ± SD (−3.275% to +2.065%) limits of agreement, as seen in Figure 3B. There was contribution to the negative bias seen with Set 2&3B, as when comparing the Bland-Altman plots between the mean BMF values acquired on Set 1B with those acquired on Set 1A, as shown in Supplementary Figure 2, there is a positive bias, suggesting that rater B's annotations tended to estimate higher mean BMF values than rater A's annotations. Despite only being trained on rater A's annotations, the U-Net segmentation provides a middle ground between the two raters, resulting in what appears to be overestimation in comparison with rater A's annotations, and underestimation in comparison with rater B's.

### Workflow Reliability

After training, the automated segmentation method is a deterministic model that returns the same segmentations (and thus BMF fractions from each lumbar vertebral body) given the input is the same IDEAL image set from the same subject, allowed for within-subject repeatability on the same scanned images. Within-subject repeatability on different scans of the same subject was also measured on a separate control dataset for a total of *n* = 8 subjects, with an overall mean BMF fraction of 33.1%. Between each lumbar vertebral body for two repeated scans, there was a precision error of 1.6% and ICC of 1.00, showing good correlation and lack of significant difference between the test-retest scans. Detailed analyses for each lumbar vertebral body are shown in Supplementary Table 2.

## Discussion

We developed, trained, and validated a deep learning model on a total of 57 IDEAL image data sets to automatically segment lumbar vertebral bodies from water-fat MRI data. Our main findings were (1) deep learning-based automatic segmentation of vertebral bodies was feasible in 16 ± 1 s; (2) the deep learning model segmented vertebral bodies with high accuracy (97.8%), precision (98.3%), and sensitivity (99.3–99.4%) when compared with manual segmentations; (3) the deep learning model showed good performance compared with standard manual analysis (mean DSC = 0.849 across 24 subjects); (4) the automatically segmented ROIs provided reliable quantification of bone marrow fat fraction (Bland Altman analysis: low bias and limits of agreements lower than 10% difference of the mean ground truth values); and (5) the automatic segmentation and BMF quantification workflow is highly repeatable between scans within the same subjects (precision error of 1.6% and ICC of 1.00). While only lumbar vertebrae were analyzed in our study, if other vertebral bodies in the thoracic or sacral regions were present in the field of view, these would also get segmented by our deep learning framework, suggesting applications for segmentation of other vertebral regions with bone marrow fat. However, further work would be required for the trained model to be generalizable for bone sites other than vertebrae, such as hips or knees, as the current model is trained to be highly sensitive to the anatomy of vertebral bodies from sagittal spine scans.

Our deep learning model was slightly lacking in performance compared to other automated vertebral segmentation methods that were used for 3D modeling from spine CT or clinical MR images. In particular, using a U-Net, Lu et al. (2018) achieved a mean DSC of 0.93 ± 0.02 when inputting higher resolution sagittal and axial T2-weighted MR images for segmenting and labeling 6 intervertebral disc levels after training on 4,075 patients (23). Their method however focused on a multi-class approach for stenosis grading, using curve fitting and bounding boxes to segment vertebral bodies rather than tight contours as ground truth. Our ground truth masks more accurately represent the true vertebral body shape in comparison, providing a more detailed and complex segmentation problem. Nevertheless, when applying our predicted segmentations for the purpose of BMF fraction quantification on lower resolution IDEAL MRI data, our Bland-Altman analysis showed performance on par with our manual segmentations. Additionally, the relatively quick process of automatic segmentation (16 ± 1 s) allows for a more efficient workflow compared to that of manual segmentation.

Our study has some limitations. Manual segmentation of the vertebrae can be very challenging depending on the pathologies present in the image. Thus, even between the two “ground truths,” there was still variability present between the two annotators as shown by the only moderately high Cohen kappa of 0.798. Nevertheless, by using multiple annotators in our study, with a neural network trained on one rater's annotations, there is better representation for real world use cases, such as in multi-center studies where other institutions would have had different annotators operating under different guidelines. Therefore, in this study, we have tested the relevance of a trained U-Net based on images annotated by a single rater against those annotated by an unseen rater.

Additionally, our method suffers from slightly lower specificity (78.2–80.2%), indicating higher incidence of false-positive labels when compared to manual segmentations. This may be due to manual segmentation guidelines preferring to underestimate the extent of the vertebral bodies in order to prevent partial volume effects. This may affect the validity of the automatically segmented vertebral bodies, although the clinical consequences of the lower specificity remain unclear since the resultant mean BMF values showed excellent Bland-Altman agreement between automated and manual analyses. In future applications, the automatic segmentation could thus be used in a semi-automatic approach, where the resulting automatic predictions can then be modified manually, still resulting in an acceleration in the workflow compared to completely manual segmentation. It is also worth acknowledging that the MRI data were acquired at a single site with the same magnet, and thus, the performance of the automatic segmentation method may need to be measured on images obtained with MRIs from different vendors. We aim to improve the performance of the neural network by utilizing data augmentation to account for the relatively small dataset, fine-tuning the data with newly acquired data and manual segmentations, and exploring alternative networks (e.g., 3D neural network architectures).

In conclusion, our study demonstrated the feasibility of an automated pipeline to segment vertebral bodies from water-fat IDEAL MR images and showed that its performance was similar to that of manual segmentation. In the future, we also plan to implement these segmentations in an automated pipeline for measuring vertebral BMF as an imaging biomarker of vertebral abnormalities, as the trained model may be useful in large studies of low back pain patients.

## Data Availability Statement

The datasets presented in this article are not readily available because data availability is restricted due to ethical considerations. Requests to access the datasets should be directed to Jiamin Zhou, jiamin.zhou@ucsf.edu.

## Ethics Statement

The studies involving human participants were reviewed and approved by UCSF Institutional Review Board. The patients/participants provided their written informed consent to participate in this study.

## Author Contributions

JZ, AF, and RK contributed conception and design of the study. JZ and RC organized the database. JC and AB performed the manual segmentations. JZ, AL, and RC performed the statistical analysis. JZ wrote the first draft of the manuscript. PD, AF, TL, and RK wrote sections of the manuscript. All authors contributed to manuscript revision, read and approved the submitted version.

## Conflict of Interest

The authors declare that the research was conducted in the absence of any commercial or financial relationships that could be construed as a potential conflict of interest.

## Supplementary Material

Supplementary Figure 1 **(A)**. Receiver operating characteristic (ROC) curves for the classification of vertebrae in training dataset Set 1A (blue), and testing datasets Set 2A (orange), and Set 2B&3B (green). Chance performance is shown by the dotted red line. Area under the receiver operating characteristic curve (AUC) was highest for the training set (0.99), as expected, but also reasonably high for both test sets (0.91 and 0.92 for **A and B**, respectively). **(B)** Precision-recall curve for the classification of vertebrae in training dataset Set 1A (blue), and testing datasets Set 2A (orange), and Set 2B&3B (green). The F1-score and area under the curve (AUC) was highest for the training set (0.98), as expected. The AUCs for both test sets are fairly high (0.87 and 0.88 for **A and B**, respectively), though lower than in the ROC curve, likely due to data imbalance.

Supplementary Figure 2 Bland-Altman plot of mean bone marrow fat fraction (BMF) percentages (%) as determined by rater B's annotations on the training dataset (Set 1B, *n* = 53 vertebrae) compared to rater A's annotations on the same subjects (Set 1A, *n* = 53 vertebrae) for each lumbar vertebral body (L1-L5). The bias was +1.352% with limits of agreement of −1.184% and +3.887%.

Supplementary Table 1 Overview of deep learning model performance results on the two tests sets used for evaluation.

Supplementary Table 2 Repeatability analyses applying a linear mixed model with random intercept to account for the repeated measured data for each lumbar vertebral body across all patients in the repeatability data set (*n* = 8).
