# Supplementary figures and images for: Automatic Vertebral Body Segmentation Based on Deep Learning of Dixon Images for Bone Marrow Fat Fraction Quantification
Source: Front Endocrinol (Lausanne). 2020 Sep 2;11:612. doi: 10.3389/fendo.2020.00612 (PMC7492292; doi:10.3389/fendo.2020.00612)

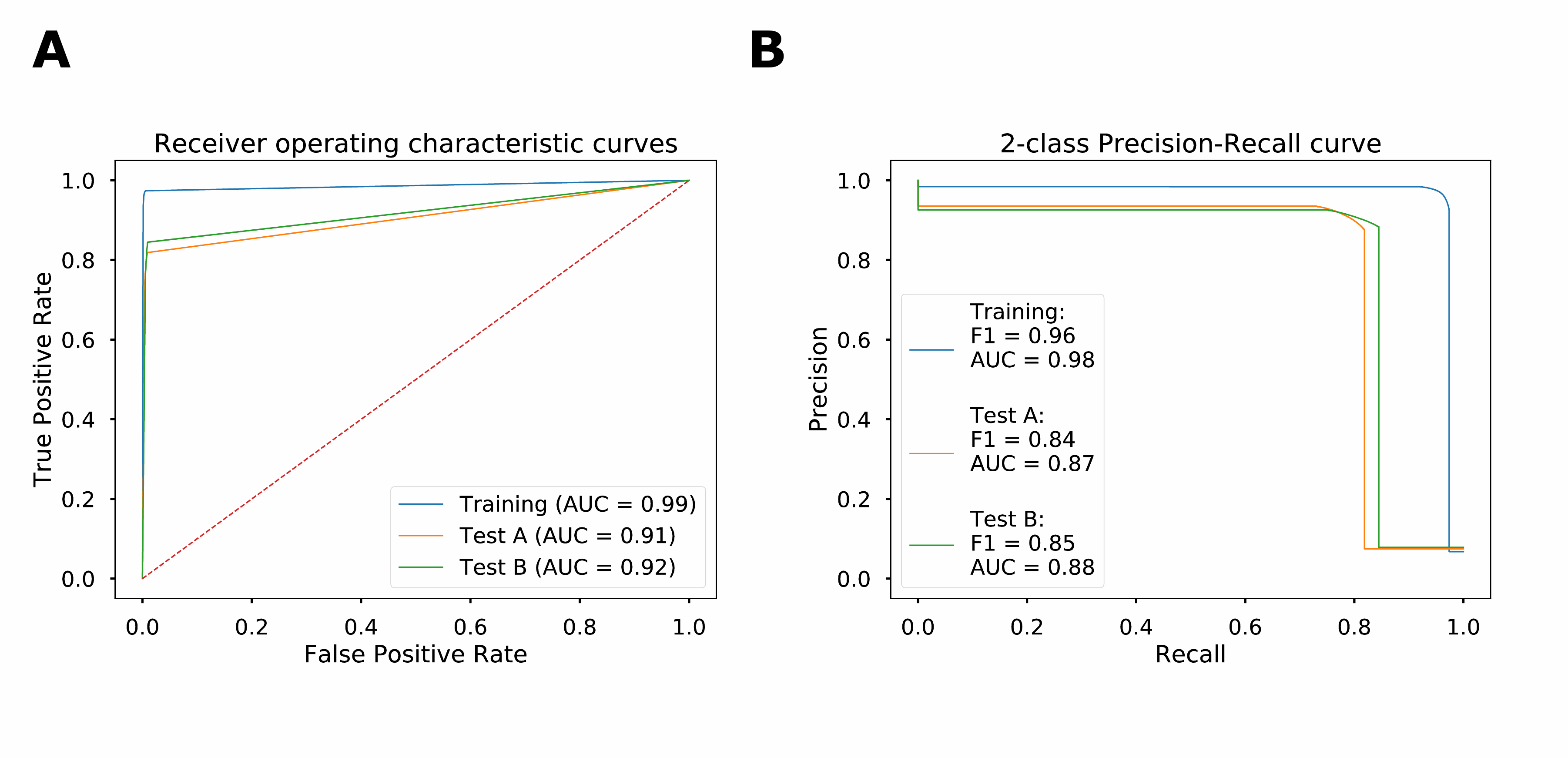

Supplement: Supplementary Figure 1 — (A). Receiver operating characteristic (ROC) curves for the classification of vertebrae in training dataset Set 1A (blue), and testing datasets Set 2A (orange), and Set 2B&3B (green). Chance performance is shown by the dotted red line. Area under the receiver operating characteristic curve (AUC) was highest for the training set (0.99), as expected, but also reasonably high for both test sets (0.91 and 0.92 for A and B, respectively). (B) Precision-recall curve for the classification of vertebrae in training dataset Set 1A (blue), and testing datasets Set 2A (orange), and Set 2B&3B (green). The F1-score and area under the curve (AUC) was highest for the training set (0.98), as expected. The AUCs for both test sets are fairly high (0.87 and 0.88 for A and B, respectively), though lower than in the ROC curve, likely due to data imbalance. [file Image_1.TIF]

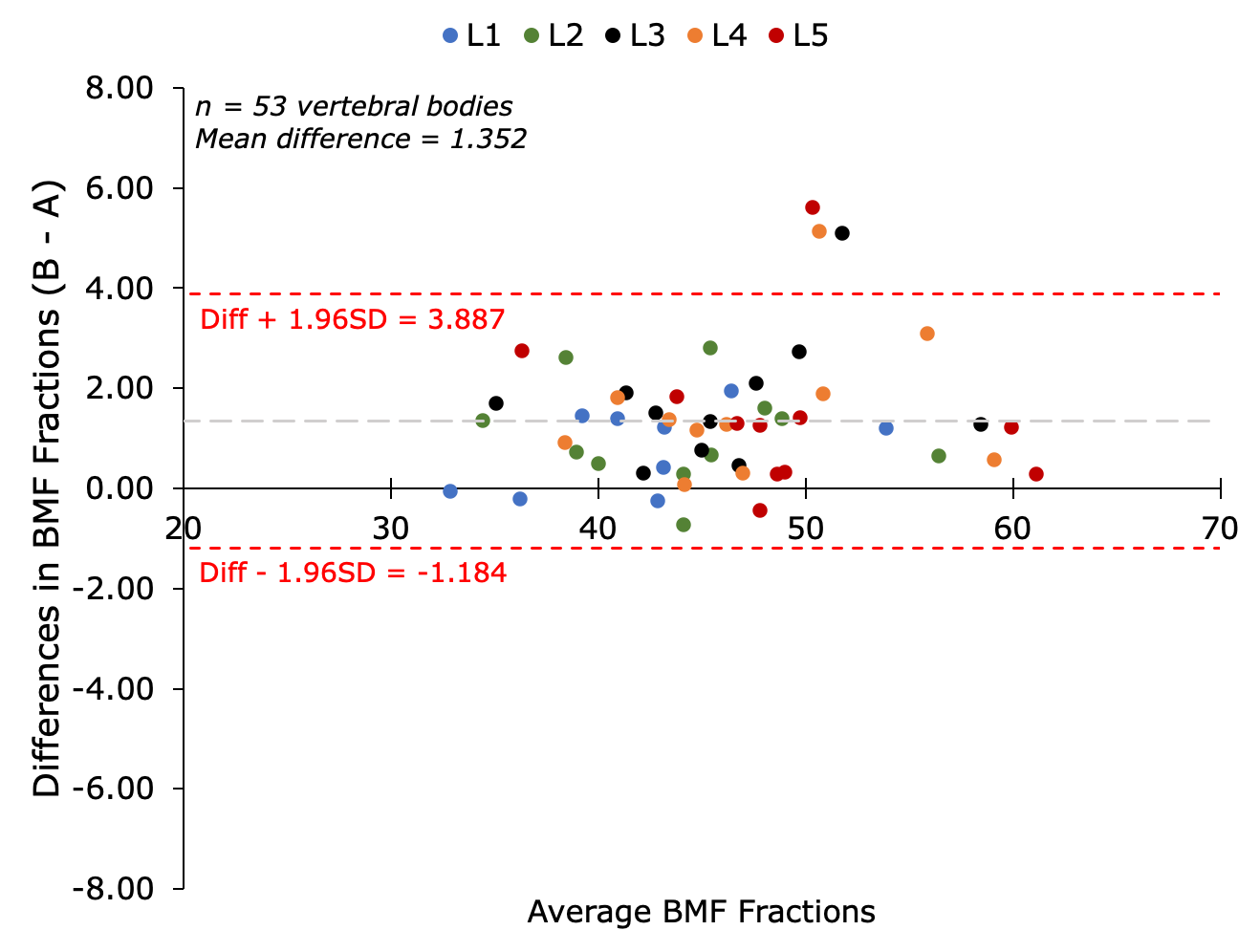

Supplement: Supplementary Figure 2 — Bland-Altman plot of mean bone marrow fat fraction (BMF) percentages (%) as determined by rater B's annotations on the training dataset (Set 1B, n = 53 vertebrae) compared to rater A's annotations on the same subjects (Set 1A, n = 53 vertebrae) for each lumbar vertebral body (L1-L5). The bias was +1.352% with limits of agreement of −1.184% and +3.887%. [file Image_2.TIFF]
