# Supplementary material for: Automatic Vertebral Body Segmentation Based on Deep Learning of Dixon Images for Bone Marrow Fat Fraction Quantification
Source: Front Endocrinol (Lausanne). 2020 Sep 2;11:612. doi: 10.3389/fendo.2020.00612 (PMC7492292; doi:10.3389/fendo.2020.00612)
Supplement: Supplementary Table 1 — Overview of deep learning model performance results on the two tests sets used for evaluation. [file Table_1.DOCX]

**Supplementary Table 1.** Overview of deep learning model performance results on the two tests sets used for evaluation.

| Performance Measures | Set 1A | Set 2A | Set 2B&3B |
| --- | --- | --- | --- |
| Accuracy (%) | 99.5 | 97.8 | 97.8 |
| Precision (%) | 99.7 | 98.3 | 98.3 |
| F1 Score (%) | 96.4 | 84.2 | 85.2 |
| Sensitivity (%) | 99.8 | 99.4 | 99.3 |
| Specificity (%) | 95.9 | 78.2 | 80.2 |

Note. –Datasets used are as described in **Figure 2**. Model predictions for each slice were binarized by a threshold of >0.5. Precision is defined as true-positive pixels divided by the sum of false-positives and true-positives. Recall (also known as sensitivity) is defined as true-positive pixels divided by the sum of true-positives and false-negatives. F1 score is defined as the harmonic mean of precision and recall.
