# Supplementary material for: Automatic Vertebral Body Segmentation Based on Deep Learning of Dixon Images for Bone Marrow Fat Fraction Quantification
Source: Front Endocrinol (Lausanne). 2020 Sep 2;11:612. doi: 10.3389/fendo.2020.00612 (PMC7492292; doi:10.3389/fendo.2020.00612)
Supplement: Supplementary Table 2 — Repeatability analyses applying a linear mixed model with random intercept to account for the repeated measured data for each lumbar vertebral body across all patients in the repeatability data set (n = 8). [file Table_2.DOCX]

**Supplementary Table 2.** Repeatability analyses applying a linear mixed model with random intercept to account for the repeated measured data for each lumbar vertebral body across all patients in the repeatability data set (*n* = 8).

| Scan Type (*n*=8) | Mean Fat Fraction (%) | 95% Confidence Interval | p-value |
| --- | --- | --- | --- |
| L1 |  |  | 0.37 |
| Test | 34.2146 | (26.5158- 41.9133) |  |
| Retest | 34.7822 | (27.0770- 42.4785) |  |
| L2 |  |  | 0.053 (0.64 when adjusted by Tukey-Kramer) |
| Test | 34.5424 | (26.8463- 42.2386) |  |
| Retest | 35.5519 | (27.8552- 43.2487) |  |
| L3 |  |  | 0.96 |
| Test | 36.0469 | (28.3526- 43.7411) |  |
| Retest | 36.0215 | (28.3269- 43.7162) |  |
| L4 |  |  | 0.70 |
| Test | 36.0463 | (28.3534- 43.7391) |  |
| Retest | 36.1991 | (28.5055- 43.8926) |  |
| L5 |  |  | 0.30 |
| Test | 36.9305 | (29.2353- 44.6258) |  |
| Retest | 37.5071 | (29.8078- 45.2064) |  |

Note. – L1-5 represent the lumbar vertebral regions of interest. A linear mixed model with random intercept was used to account for the repeated measured data. To compare the mean fat fraction percentages, a repeated-measures ANOVA was used with a least mean-squares method. A *post hoc* t-test was used to evaluate the difference in the first and second fat fraction percentages taken from the scans.
